# A Judging Scheme for Large-Scale Innovative Class Competitions Based on Z-Score Pro Computational Model and BP Neural Network Model

**DOI:** 10.3390/e27060591

**Published:** 2025-05-31

**Authors:** Zhuoting Yu, Hongzhong Deng, Shuaiwen Tang

**Affiliations:** College of Systems Engineering, National University of Defense Technology, Changsha 410073, China; yuzhuoting19@nudt.edu.cn (Z.Y.); tsw631845201@163.com (S.T.)

**Keywords:** large-scale innovation competition, optimization of review plan, genetic algorithm, Z-score model, BP neural network, information theory

## Abstract

Recently, interest in optimizing judging schemes for large-scale innovation competitions has grown as the complexities in evaluation processes continue to escalate. Although numerous methods have been developed to improve scoring fairness and precision, challenges such as evaluator subjectivity, workload imbalance, and the inherent uncertainty of scoring systems remain inadequately addressed. This study introduces a novel framework that integrates a genetic algorithm-based work cross-distribution model, advanced Z-score adjustment methods, and a BP neural network-enhanced score correction approach to tackle these issues. First, we propose a work crossover distribution model based on the concept of information entropy. The model employs a genetic algorithm to maximize the overlap between experts while ensuring a balanced distribution of evaluation tasks, thus reducing the entropy generated by imbalances in the process. By optimizing the distribution of submissions across experts, our model significantly mitigates inconsistencies arising from diverse scoring tendencies. Second, we developed modified Z-score and Z-score Pro scoring adjustment models aimed at eliminating the scoring discrepancies between judges, thereby enhancing the overall reliability of the normalization process and evaluation results. Additionally, evaluation metrics were proposed based on information theory. Finally, we incorporate a BP neural network-based score adjustment technique to further refine the assessment accuracy by capturing latent biases and uncertainties inherent in large-scale evaluations. Experimental results conducted on datasets from national-scale innovation competitions demonstrate that the proposed methods not only improve the fairness and robustness of the evaluation process but also contribute to a more scientific and objective assessment framework. This research advances the state of the art by providing a comprehensive and scalable solution for addressing the unique challenges of large-scale innovative competition judging.

## 1. Introduction

In the era of rapid technological innovation, a growing number of innovation-oriented competitions have emerged as vital platforms for cultivating modeling and creative problem-solving skills. Several prominent examples of innovation-oriented competitions include the “Internet Plus” Innovation and Entrepreneurship Competition targeting Chinese university students [1], the internationally recognized Mathematical Contest in Modeling [2], and various technology R&D innovation challenges initiated by corporate entities [3,4,5]. These competitions are characterized by their wide participant base, complex subject matter, high interdisciplinarity, and lack of standard answers—features that not only enhance their educational value but also present significant challenges for fair and consistent evaluation.

Due to the subjective nature of human judgment and the inherent ambiguity in evaluating open-ended tasks, the assessment of competition submissions is prone to issues such as inconsistent evaluation standards, strong evaluator subjectivity, and limited robustness of scoring methods [6,7]. In large-scale competitions with multi-stage review processes (typically including online review, on-site evaluation, and oral defense), the uncertainty and entropy of the evaluation process tend to increase. Variations in individual expert judgments may introduce systemic noise, especially when the difference in expert scores (i.e., score range) is substantial.

Existing evaluation schemes attempt to mitigate these inconsistencies using standardization, score trimming (e.g., removing the highest and lowest scores), or expert consensus discussions [8,9]. In order to reduce systematic biases, researchers often adopt the standardization approach [10], which involves transforming each expert’s scores such that their mean and variance are normalized. The underlying assumption is that the submissions evaluated by each expert follow an identical quality distribution. However, this assumption may not hold in practice, as the actual quality of submissions reviewed by different experts can vary significantly. Consequently, in scenarios where it is impractical for all experts to score every submission, the use of standardized scoring methods may still introduce considerable errors. Johnson et al. [11] introduced a multistage enhancement approach to scoring; Urbano et al. [12] have highlighted the potential of score normalization techniques in the evaluation of information retrieval (IR) systems, especially when working with test collections. Their work emphasizes that standardization can help ensure fair comparisons across different system collections while mitigating the impact of anomalous results on individual topics. Building on this, Sakai [13] proposed a method that transforms raw scores into standardized values by utilizing the topic-wise mean and standard deviation derived from historical system runs. This enables each system’s performance to be interpreted in terms of how much it deviates, in standard deviation units, from the average performance across runs, offering a normalized and robust evaluation framework. Guo et al. [14] proposed a weighted and improved Z-score method. However, these approaches have limited scalability and may not fully address the inherent complexity and entropy of large-scale competition systems. As the number of participants and evaluators increases, the scoring process itself becomes a high-entropy system, where fairness, reliability, and reproducibility become key concerns.

Under these conditions, it is necessary to establish a procedural evaluation model for the early stages of the review process. The BP (Back Propagation) neural network, introduced in 1986 by Rumelhart et al., is a multilayer feedforward neural network trained using the error BP algorithm. It is one of the most widely used neural network models [15]. The BP neural network is a multilayer feedforward network trained using the error BP algorithm. The core idea behind this algorithm is gradient descent, which utilizes gradient search techniques to minimize the mean squared error between the network’s actual output and the desired output [16,17]. Due to its excellent performance, as well as its ability to classify patterns of arbitrary complexity and map multidimensional functions effectively, it is widely used in various fields [18,19,20,21,22]. In terms of evaluation, Liu et al. [23] proposed a new method for assessing undergraduate education quality based on the BP algorithm and stress testing; Zhang et al. [24] developed a novel temporal adaptive fuzzy neural network for fatigue detection based on facial features. The BP neural network has demonstrated strong performance in both evaluation and prediction tasks; however, to the best of our knowledge, it has not yet been applied to the evaluation of large-scale competitions.

In this context, it is essential to design programmatic evaluation frameworks that are not only statistically rigorous but also resilient to the influence of extreme judgments and systematic noise. This paper addresses the challenge by analyzing a two-stage dataset from a national-scale innovation competition. In the first stage, five experts independently score each submission using standardized metrics, and top-performing works are shortlisted. In the second stage, three different experts re-evaluate the shortlisted submissions, and necessary adjustments are made to extreme scores to ensure robustness. Final scores are computed as the average of both stages’ standardized ratings. Building on this dataset, we propose a novel evaluation scheme based on the Z-score Pro model and a BP neural network to better capture latent patterns in expert assessments and to reduce entropy within the ranking process. The results demonstrate the potential of intelligent modeling to improve the fairness, consistency, and scientific validity of evaluations in high-stakes, large-scale innovation competitions.

## 2. Models and Methods

### 2.1. Cross-Distribution of Works Based on Genetic Algorithms

When constructing the cross-distribution model for works, it is necessary to maximize the overlap of reviewed works between every pair of experts while ensuring that the number of works assigned to each expert and the degree of overlap remain relatively balanced. From an information-theoretic standpoint, the cross-distribution scheme can be viewed as a process of maximizing the information entropy of review coverage while maintaining controlled overlap among reviewers. Entropy, a fundamental concept in information theory, quantifies the uncertainty or diversity of a system. In this context, a higher entropy value in the review distribution implies greater diversity in reviewer combinations across submissions, which enhances the robustness of evaluation and mitigates the bias caused by reviewer-specific tendencies. Mathematically, the entropy *H* of the distribution of submissions among reviewers can be expressed as:

(1) $$H=-\sum_{i=1}^{n}\sum_{k=1}^{m}p_{ik}\log p_{ik}$$

where $p_{ik}$ denotes the probability that reviewer *i* reviews submission *k*, derived from the binary matrix $x_{ik}$. Maximizing this entropy, subject to workload balance and overlap constraints, helps ensure an optimal review assignment that captures a broad spectrum of perspectives.

Based on these requirements, we developed a cross-distribution model for works and employed a genetic algorithm to solve for the optimal distribution scheme.

#### 2.1.1. Model for Cross-Distribution of Works

Let *m* be the total number of participating teams in the competition, where each team is allowed to submit only one entry. The entries are numbered from 1 to *m*. The competition is evaluated by *n* judges, each identified by a unique number from 1 to *n*. Each entry is assessed by a subset of *t* judges who provide their scores. The cross-distribution model for entries is established as follows:

(2) $$\min z=\left(\max Q_{ij}-\min Q_{ij}\right)$$

(3) $$\;s.t.\;\left\{\begin{array}{c} d_{i}\leq \sum_{k=1}^{m}x_{ik}\leq u_{i}\\ \sum_{i=1}^{n}x_{ik}=t\\ Q_{ij}=\sum_{k=1}^{m}\left(x_{ik}\wedge x_{jk}\right)\\ x_{ik}=0\;or\;1 \end{array}\right.$$

where $x_{ik}=0$ or 1, with $x_{ik}=1$ indicating that the *k*-th entry is reviewed by the *i*-th judge; otherwise, $x_{ik}=0$. $u_{i}$ and $d_{i}$ represent the maximum and minimum number of reviews assigned to judge *i*, respectively. $Q_{ij}$ denotes the number of entries assigned to both judges *i* and *j*. $d_{i}\leq \sum_{k=1}^{m}x_{ik}\leq u_{i}$ represents the constraint on the number of entries reviewed by judge *i*. By adjusting the values of $u_{i}$ and $d_{i}$, the workload among judges can be balanced as evenly as possible. $\sum_{i=1}^{n}x_{ik}=t$ ensures that each entry is reviewed by exactly *t* judges. $Q_{ij}$ denotes the number of entries assigned to both judges *i* and *j*, expressed as $Q_{ij}=\sum_{k=1}^{m}\left(x_{ik}\wedge x_{jk}\right)$. Here, $x_{ik}=0$ or 1, where $x_{ik}=1$ indicates that judge *i* reviews entry *k*. If $x_{ik}=x_{jk}=1$, then $x_{ik}\wedge x_{jk}=1$, meaning that entry *k* is reviewed by both judges *i* and *j*; otherwise, $x_{ik}\wedge x_{jk}=0$.

#### 2.1.2. Genetic Algorithm Based Cross-Distribution Scheme Solution for Works

We employ a genetic algorithm to solve the work crossover distribution problem. The workflow of the Algorithm 1 is as follows:
**Algorithm 1:** Cross-Distribution Algorithm-Based on Genetic Algorithm
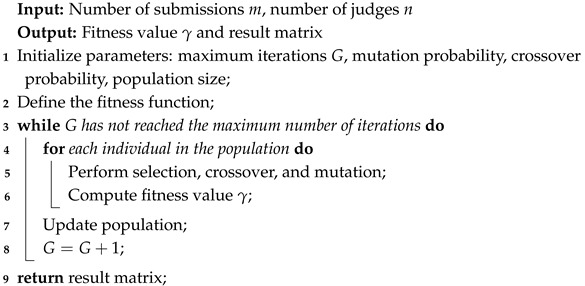


### 2.2. Score Adjustment Model

In current peer review processes, ranking methods based on standardized scores (Z-scores) are commonly employed. These methods rest on the assumption that the distribution of academic quality is consistent across the sets of submissions evaluated by different reviewers. However, in large-scale innovation competitions, each submission is typically reviewed by only a small number of experts, and there is limited overlap between the works assessed by any two reviewers. Consequently, each expert evaluates only a small subset of the total submissions, and the assumption of a uniform distribution of academic quality may no longer hold. This limitation calls for the development of alternative evaluation models.

In this section, we focus on the review process for large-scale innovation competitions and propose a modified Z-score model, building upon the conventional standardization approach. Furthermore, considering that the final ranking of first-prize submissions in the second stage of evaluation is achieved through expert consensus, we leverage this information to develop the Z-score Pro model. To assess the effectiveness of the proposed methods, we also introduce four evaluation metrics.

#### 2.2.1. Z-Score Model

Z-score, also known as the standard score or zero-mean normalization, is a widely used statistical tool for representing the distance or deviation of an observation or data point from the mean. Expressed in units of standard deviation, it reflects how far a given score deviates from the average in a relative and standardized manner.

To compute a Z-score, the difference between the raw data point and the mean is divided by the standard deviation. This calculation yields a Z-score that quantifies the distance of a specific score from the mean, measured in standard deviation units. The specific procedure is as follows:

(1) For a given reviewer, let the assigned scores be represented by a set of *n* samples: $a_{1},a_{2},\ldots ,a_{n}$.
(2) The sample mean of the scores given by this reviewer is calculated as:
  (4) $$\bar{a}=\frac{1}{n}\sum_{k=1}^{n}a_{k}$$
(3) The sample standard deviation of the reviewer’s scores is given by:
  (5) $$s=\sqrt{\frac{1}{n-1}\sum_{k=1}^{n}{\left(a_{k}-\bar{a}\right)}^{2}}$$
(4) The Z-score is then computed as:
  (6) $$x_{k}=50+10\times \frac{a_{k}-\bar{a}}{s}$$

In Equation (6), the constants 50 and 10 control the mean and amplitude of the standardized scores, respectively. Since this standardization formula is predefined and adopted in the original problem, we do not further discuss the rationale behind the selection of these specific values. To ensure the comparability between our proposed model and the original standardization method, we retain the use of these two constants in the subsequent modeling process.

The final score of submissions that participate only in the first-stage review is calculated as the average of the standardized scores assigned by five reviewers. Submissions are then ranked based on this average. For submissions that undergo both the first and second stages of review, the final score consists of two parts: (1) the average of the standardized scores from the first-stage review, and (2) the sum of three standardized scores derived from the raw scores given by three reviewers in the second-stage review, after appropriate adjustments. The final ranking for these submissions is determined based on the combined score from both stages.

#### 2.2.2. Modified Z-Score Model

In scoring systems, entropy can be used to measure the dispersion and uncertainty of the score distributions across submissions. Before standardization, raw scores typically exhibit varying ranges and biases among reviewers. The Z-score model implicitly reduces entropy by normalizing scores to a common distribution, which facilitates comparability but may lead to information loss—especially when outlier scores reflect meaningful expert judgments.

Given a set of scores $a_{1}$, $a_{2}$, …, $a_{n}$ from a reviewer, the normalized Shannon entropy is given by:

(7) $$H=-\sum_{j=1}^{k}p_{j}\log p_{j}$$

where $p_{j}$ is the empirical probability of score level *j*. High entropy indicates high diversity in scoring, while low entropy suggests convergence or bias. By comparing entropy levels before and after normalization, we can quantify the extent to which the standardization process simplifies (or distorts) the underlying evaluation signal.

To objectively determine which scores qualify as extreme and require adjustment, we apply the interquartile range (IQR) method. Specifically, any score that falls below $Q_{1}-1.5\times IQR$ or above $Q_{3}+1.5\times IQR$, where $IQR=Q_{3}-Q_{1}$, is identified as an outlier. This statistical rule ensures that the adjustment process is data-driven and minimizes subjective threshold selection.

However, the traditional Z-score method has several limitations. First, the calculation of Z-scores requires knowledge of the population mean and variance, which are often difficult to obtain in real-world analysis and data mining scenarios. In most cases, the sample mean and standard deviation are used as substitutes. Second, the Z-score method assumes certain characteristics of the data distribution, with normal distribution being the most favorable condition for its application. Third, the Z-score transformation removes the original meaning of the data. The Z-scores of items A and B are no longer directly related to their original scores. As a result, Z-scores are only useful for relative comparison between data points, while the actual values must be recovered to interpret the real-world meaning. Moreover, both the mean and standard deviation are sensitive to outliers, which may distort the standardized results and compromise the balance of feature scaling. To address these issues, we propose a modified Z-score method that reduces the sensitivity to outliers and minimizes potential distortions.

To overcome the sensitivity of the traditional Z-score method to the mean and standard deviation, and to prevent large deviations caused by outliers, we propose a modified Z-score method. The general form of the modified Z-score method is defined as:

(8) $$Z_{i}=\frac{x_{i}-\mathrm{median}\left(x_{i}\right)}{MAD}$$

where $x_{i}$ represents the sample score, $\mathrm{median}(x_{i})$ is the median of all sample observations, and MAD (Median Absolute Deviation) is defined as:

(9) $$MAD=\mathrm{median}\left|x_{i}-\tilde{x_{i}}\right|$$

The standard deviation is based on the sum of squared distances from the mean, making it highly sensitive to outliers. For example, a large sample value within the dataset directly affects the standard deviation. In contrast, MAD remains unaffected by such extreme values, offering better robustness.

In this paper, the formula for the modified Z-score model is specified as:

(10) $$Z_{i}=50+10\times \frac{x_{i}-\mathrm{median}\left(x_{i}\right)}{MAD}$$

#### 2.2.3. Z-Score Pro Model

The modified Z-score method reduces the influence of outliers on the results, but it is essentially the same as the traditional standard score model. Both methods use statistical measures from the sample data to fit the original scores, aiming to approximate a reasonable distribution. This approach is quite reasonable for general cases; however, when applied to large-scale innovation competitions, it overlooks an important factor—the range (or “extreme values”). The size of the range is non-linearly related to the creativity of the submissions. Therefore, by incorporating the ranking of first-prize submissions, which was determined through expert consensus, we propose an improvement to the modified Z-score method, leading to the introduction of the Z-score Pro model.

In this study, the second stage of review involves three experts evaluating the submissions. The standardized scores are calculated for each submission, and necessary adjustments are made for those with large ranges. Then, the average of the five experts’ standardized scores from the first stage and the three experts’ standardized scores from the second stage are summed. The final ranking is determined based on this total score, as follows:

(1) For submissions with small ranges that were not re-evaluated, the total score is:
  (11) $$x_{c}=\bar{x_{i}}+\sum_{q=1}^{3}x_{iq}$$
  where $x_{c}$ represents the final total score, $\bar{x_{i}}$ is the average of the first stage standardized scores for submission *i* from five experts, and $x_{iq}$ represents the standardized score for submission *i* given by the *q*-th expert in the second stage.
(2) For submissions with large ranges that were re-evaluated, the total score is:
  (12) $$x_{c}=\bar{x_{i}}+\sum_{q=1}^{3}f_{iq}$$
  where $x_{c}$ represents the final total score, $\bar{x_{i}}$ is the average of the first stage standardized scores for submission *i*, and $f_{iq}$ represents the re-evaluated score for submission *i* from the *q*-th expert in the second stage.

The total score using the modified Z-score method is calculated by summing the average of the first stage standardized Z-scores from five experts, and the standardized Z-scores from three experts in the second stage, as follows:

(13) $$Z_{c}=\bar{Z_{i}}+\sum_{q=1}^{3}Z_{iq}$$

where $Z_{c}$ represents the total score using the modified Z-score method, $\bar{Z_{i}}$ is the average of the first stage standardized Z-scores for submission *i*, and $Z_{iq}$ represents the standardized Z-score for submission *i* given by the *q*-th expert in the second stage.

For the first-prize submissions, the rankings are determined by expert consensus and considered as precise data. Using data from 27 first-prize submissions, the impact of the range on the rankings is considered, and a quadratic function fitting is applied to improve the model. The procedure is as follows:

(1) Compute the difference:
  (14) $$C=x_{c}-Z_{c}$$
  where *C* represents the difference between the adjusted score ($x_{c}$) and the modified Z-score-based score ($Z_{c}$).
(2) To capture the non-linear effect of score range *R* on this deviation, we performed a curve fitting procedure using multiple candidate models, including linear, logarithmic, exponential, and polynomial functions. Among them, the quadratic function exhibited the best balance between fit accuracy and interpretability. Specifically, it achieved the highest $R^{2}$ value (0.87) and showed clear parabolic characteristics in the residual distribution, whereas other forms either underfit the data or produced unstable coefficients. Therefore, we fit the deviation *C* to a quadratic function of the range *R*, yielding the parameters *m*, *n*, and *o*:
  (15) $$C=mR^{2}+nR+o$$
  The calculated values are m=−0.055, n=1.736, and o=−13. This quadratic form reflects the observation that extremely low or high ranges have non-proportional effects on ranking bias, aligning with the empirical distribution of creative submission scores.
(3) The formula for calculating the Z-score Pro score is:
  (16) $${Zp}_{i}=50+10\times \frac{x_{i}-\mathrm{median}\left(x_{i}\right)}{MAD}+\left(mR_{i}^{2}+nR_{i}+o\right)$$
  where ${Zp}_{i}$ is the Z-score Pro score, $x_{i}$ is the sample score, $\mathrm{median}(x_{i})$ is the median of all sample observations, MAD is the Median Absolute Deviation, and $R_{i}$ is the range of submission *i*.
(4) The final total score using the Z-score Pro method is calculated by summing the average of the Z-score Pro scores from five experts in the first stage and the Z-score Pro scores from three experts in the second stage, as follows:
  (17) $${Zp}_{c}=\bar{{Zp}_{i}}+\sum_{q=1}^{3}{Zp}_{iq}$$
  where ${Zp}_{c}$ represents the final total score using the Z-score Pro method, $\bar{{Zp}_{i}}$ is the average of the Z-score Pro scores from five experts in the first-stage, and ${Zp}_{iq}$ represents the Z-score Pro score for submission *i* given by the *q*-th expert in the second stage.

The Z-score Pro model relies on standardized scores derived from the mean and standard deviation of each evaluator’s score set. A constant value of 50 is used in the model because it is specifically designed for a 0–100 scoring range. If different scoring scales are used, the scores can be normalized to the [0, 100] interval through a standard normalization process, enabling the model to remain applicable across various evaluation settings.

#### 2.2.4. Evaluation Metrics

To reflect the relative advantages of each method, this study designs four evaluation metrics based on information-theoretic principles. In decision-making and evaluation systems, the core ideas of information theory can be employed to quantify uncertainty, divergence, and consistency among outcomes. Especially in contexts involving rankings or judgments, analyzing the informational differences between various ranking results allows for a more objective and nuanced assessment of how a given ranking deviates from a reference standard.

Given that the scores in the second stage were obtained after experts’ careful deliberation and reassessment, they are regarded as accurate and reliable. Therefore, in designing the evaluation metrics, we focus exclusively on the degree of deviation from these standard results, rather than assessing the methods in isolation.

(1) Overlap Degree: The number of instances where the subjective and objective rankings match is called the overlap degree, denoted as *C*.
(2) Disorder Degree: The sum of the absolute differences between the subjective and objective rankings is called the disorder degree. Denoted as *D*, it is calculated as:
  (18) $$D=\sum_{i=1}^{N}\left|t_{i}-l_{i}\right|$$
  where $t_{i}$ represents the subjective ranking of submission *i*, and $l_{i}$ represents the objective ranking of submission *i*.
(3) Divergence Degree: The Divergence degree between the two rankings is defined as:
  (19) $$\theta =\sqrt{\frac{1}{N}\sum_{i=1}^{N}{\left(t_{i}^{1}-t_{i}^{2}\right)}^{2}}$$
  where $t_{i}^{1}$ and $t_{i}^{2}$ represent the rankings of submission *i* in the two different ranking methods.
(4) Award Change Degree: This refers to the number of submissions that were awarded under the original scheme but would not be awarded under the new scheme.

To examine whether the proposed evaluation metrics—Overlap Degree, Disorder Degree, Divergence Degree, and Award Change Degree—can effectively reflect the rationality and stability of final rankings, we conducted a correlation analysis using a reference ranking constructed through the Borda count method based on consensus scores.For each experimental run, we calculated the Kendall’s Tau distance between the model-generated ranking and the reference ranking, treating it as an indicator of ranking deviation in Table 1.

The results show strong correlations between the evaluation metrics and ranking deviation. Specifically, higher overlap and lower disorder or divergence tend to indicate a closer alignment with the reference ranking. This validates the effectiveness of these metrics in assessing the fairness and rationality of the evaluation outcome.

### 2.3. A Range-Based Programmable Model Based on BP Neural Network

The defining feature of innovation-oriented competitions lies in their emphasis on creativity and the absence of standard answers. Due to the high complexity of the problems posed in such competitions, participants are generally required to propose innovative solutions to make partial progress within the competition timeframe. However, the extent of innovation demonstrated by a given submission, as well as its potential for future research, is often subject to divergent opinions. Even face-to-face discussions among experts may fail to reach consensus, as evaluators tend to hold differing perspectives. This divergence is further exacerbated by graduate students’ limited ability to articulate their work clearly and the varying evaluation criteria adopted by different judges. As a result, substantial discrepancies frequently emerge in the scores assigned to the same project by different experts.

The range, defined as the difference between the highest and lowest scores assigned to a single submission within the same evaluation stage, is a typical characteristic of large-scale innovation competitions. Submissions with large scoring ranges are often found at the upper or lower ends of the overall score spectrum. To explore the relationship between score range and innovativeness, and to identify truly innovative works among those with significant scoring discrepancies, this study introduces a programmable range model based on a BP neural network. The model is designed to address ranking optimization problems based on expert scoring data and is particularly well-suited for scenarios in which submissions in the middle-score range exhibit substantial score variability or lack evaluative consensus.

#### 2.3.1. Requirements for Constructing the Programmable Range Model

The development of the programmable range model is guided by two key requirements. First, the model should be capable of categorizing submissions based on expert scores from the initial evaluation stage. Specifically, it should be able to identify submissions with large scoring ranges and classify them accordingly. Second, the model must enable programmable processing of these categorized high-range submissions, such that adjustments can be made to their scores based on a consistent set of principles.

The overall modeling approach involves training a neural network using data obtained from the second-stage expert reassessment. By learning the relationship between standardized scores and reassessed scores in the second stage, the model captures how scores assigned by outlier experts relate to both the mean standardized score and the final reassessed score. This learned relationship is then applied to adjust the outlier expert scores from the first evaluation stage in a systematic and automated manner.

#### 2.3.2. The Programmable Range Model

**Figure 1:**
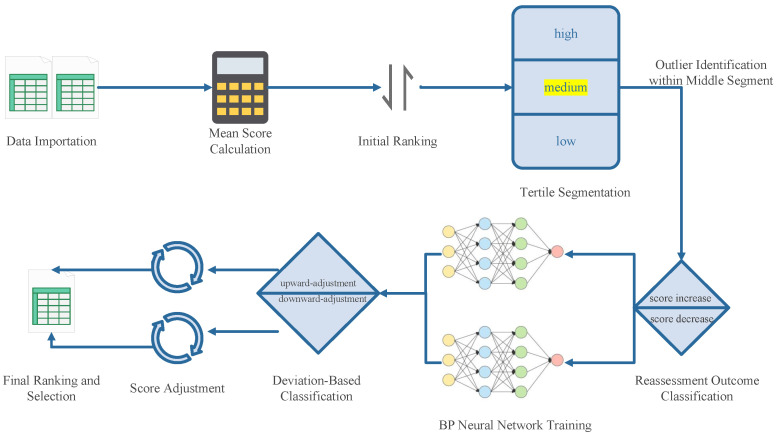
The flowchart of the Programmable Range Model.

The flowchart of the Programmable Range Model is shown in Figure 1, and the specific steps are as follows:

**Step 1: Data Importation:** Load the expert scoring data, including standardized or raw scores, along with corresponding identifiers for works and reviewers.
**Step 2: Mean Score Calculation:** For each work, calculate the average standardized score $\bar{x_{i}}$ across all experts, which serves as the basis for initial ranking.
**Step 3: Initial Ranking:** Rank all works based on their average scores $\bar{x_{i}}$ in descending order.
**Step 4: Tertile Segmentation:** Divide the ranked works into three segments: high, middle, and low. The proportion of each segment can be customized, e.g., top 15% as high, middle 50% as medium, and bottom 35% as low.
**Step 5: Outlier Identification within Middle Segment:** For each work in the middle segment, compute the score range $R_{i}$ (maximum minus minimum expert score). Define a range threshold $\bar{\bar{R}}$ and identify works with $R_{i}>\bar{\bar{R}}$ as *range-outlier candidates* for further reevaluation.
**Step 6: Reassessment Outcome Classification:** Categorize range-outlier candidates into two groups based on changes in their reassessed scores: *score increase* and *score decrease*.
**Step 7: BP Neural Network Training:** For each of the two categories (upward and downward adjustment), a separate BP neural network is trained. The independent variables include the five standardized scores provided by the experts and their mean, while the dependent variable is the re-evaluated score obtained from the second-round review. This design aims to capture the latent non-linear mapping between expert judgments and revised outcomes.

To examine the adjustment mechanism in middle-segment works with inconsistent evaluations, the data is imported into MATLAB’s Neural Network Toolbox. The dataset is randomly split into 70% for training, 15% for validation, and 15% for testing. The number of neurons in the hidden layer is set to 10, a value determined empirically through iterative testing and validation performance. This configuration ensures sufficient model capacity without overfitting, which is crucial given the moderate size of the reassessment dataset.

The training algorithm employed is the Quantized Conjugate Gradient (QCG) method, selected for its robustness and efficiency in handling regression tasks with relatively small sample sizes. The *tanh* activation function is used in the hidden layer to provide smooth, non-linear transformations, while a linear activation function is applied in the output layer to accommodate the continuous nature of the scoring outcome. This architecture enables the network to approximate the re-evaluation function effectively while maintaining stability and generalizability.

**Step 8: Deviation-Based Classification:** For each range-outlier candidate, calculate the absolute deviation of each expert’s score from the mean $\bar{x_{i}}$. Identify the expert with the largest deviation. If this expert’s score is higher than the mean, the work is classified as a *downward-adjustment* case; otherwise, it is classified as an *upward-adjustment* case.
**Step 9: Score Adjustment:** Use the trained BP neural networks to adjust the most deviant expert score for each candidate. Apply the *score-decrease network* to downward-adjustment cases and the *score-increase network* to upward-adjustment cases to generate revised scores.
**Step 10: Final Ranking and Selection:** Re-rank all works based on their adjusted scores and select a subset for advancement to the next evaluation stage according to predefined criteria.

## 3. Experiment

### 3.1. Experimental Background

This study uses data from a large-scale innovation competition evaluation, which includes 3000 participating teams and 125 review experts. Each work is evaluated by five experts, and the data contains the raw scores, standardized scores from two evaluation stages, as well as the scores after re-evaluation for works with large score discrepancies. The data consists of two stages of evaluation. In the first stage, five experts evaluate the works, calculate the standardized scores, and compute the average of these standardized scores. Based on a predetermined proportion, the top works are selected to enter the second stage. In the second stage, three experts conduct the evaluation and assign standardized scores individually. For works with extreme scores, necessary adjustments to the standardized scores are made. Finally, the standardized scores from the five experts in the first stage and the three experts in the second stage are summed and averaged. The works are then ranked based on the final total score.

### 3.2. Cross-Distribution Scheme for Works

The genetic algorithm is used to solve the established cross-distribution model for works. The initial value of the iteration count *G* is set to 0, and the termination value is set to 800. The crossover probability is 0.8, the mutation probability is 0.05, and the population size is 50. Some of the results of the solution are shown in Table 2.

**Figure 2:**
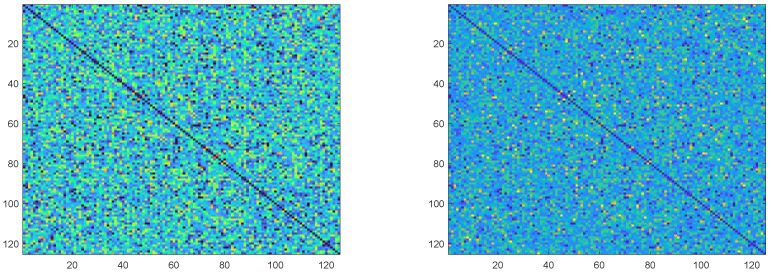
Heatmap of the crossover degree for the random distribution scheme and the cross-distribution scheme for works. The (**left**) figure represents the random distribution scheme, and the (**right**) figure represents the cross-distribution scheme for works.

To compare the effects of the cross-distribution scheme for works with the random distribution scheme, we use a heatmap to visually display the degree of crossover. The size of each unit in the heatmap is 125×125, where each unit in the matrix represents the crossover degree between two experts, i.e., the number of works evaluated by both experts. From an information-theoretic perspective, a more balanced crossover distribution implies a reduction in allocation entropy, indicating that the evaluation information is more uniformly and efficiently disseminated among experts. As shown in Figure 2, it is evident that under the cross-distribution scheme, the crossover degrees are more evenly distributed and exhibit a higher overall level, reflecting a substantial improvement in information coverage and consistency compared to the random distribution scheme.

### 3.3. Application of the Score Adjustment Models

In the methodology section, we propose the Modified Z-score calculation model and the Z-score Pro model for adjusting the scores of works. In the data, we know that the ranking of the 27 works that won the first prize in the second stage was determined after careful consideration and discussion by the experts. Therefore, we use the two proposed calculation models to compute the ranking of these 27 first-prize works and compare them with the rankings in the data. The results are shown in Table 3 and Table 4.

As mentioned earlier, a higher overlap degree is better, while a smaller disorder degree and divergence degree are preferable. Comparing the Modified Z-score and Z-score Pro methods, it is evident that the results obtained using the Z-score Pro model are superior to those of the Modified Z-score.

**Figure 3:**
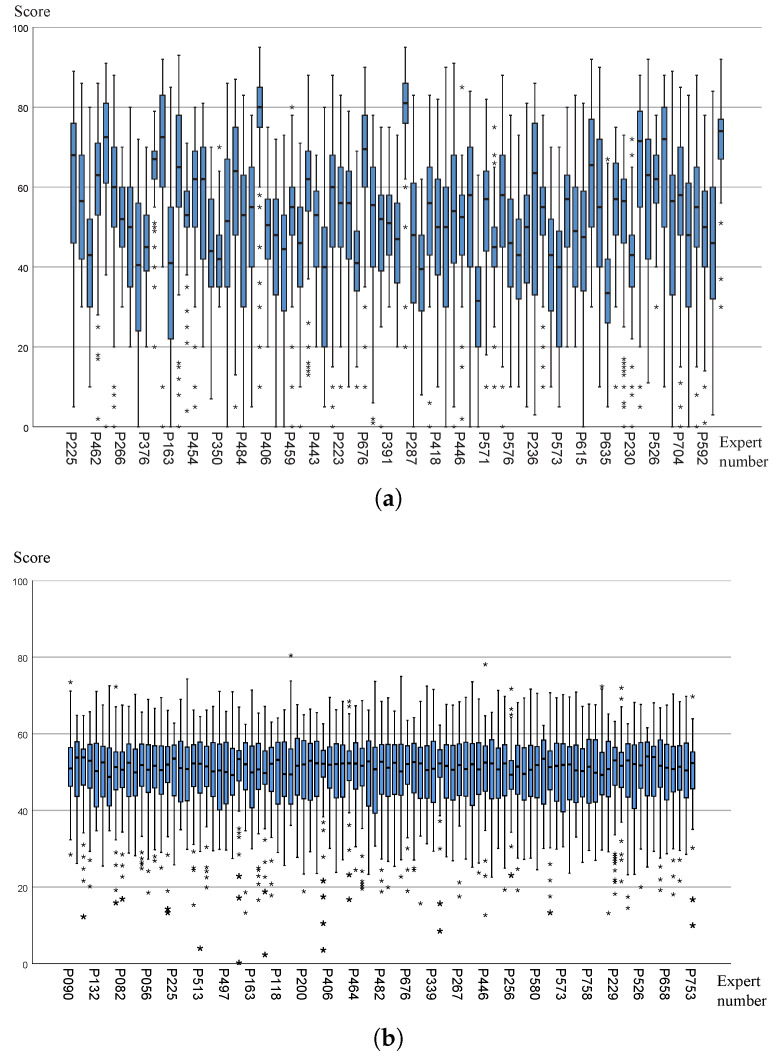
Comparison of Score Distributions Before and After Adjustment. (**a**) Raw Scores. (**b**) Adjusted Scores after Z-score Pro. * Indicates an outlier.

Furthermore, after applying the Z-score Pro standardization, the previously anomalous scores assigned by certain experts become largely aligned with the adjusted average scores of other experts, with comparable distribution ranges. This standardization not only enhances the consistency of expert judgments but also minimizes the uncertainty inherent in the original scores. From an information-theoretic perspective, the application of the Z-score Pro method reduces the entropy of the score distribution, indicating a decrease in the level of disorder or unpredictability within the expert assessments. To demonstrate this effect, we present boxplots comparing the original and standardized scores, as shown in Figure 3, which visually highlight the reduction in variability and improved alignment between expert ratings.

### 3.4. Application of the Range-Based Programmable Model Based on BP Neural Network

#### 3.4.1. Analysis of the Score and Range Variations in Two Stages

**Figure 4:**
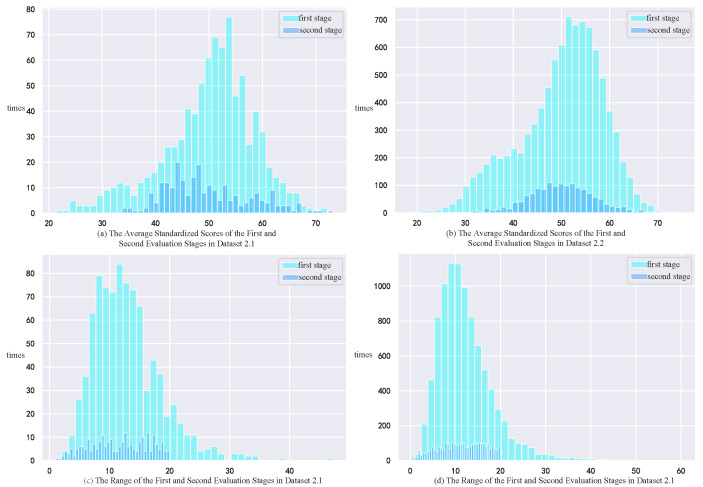
Histogram of the Changes in the Mean Standardized Score and Range.

The score data from the second stage are divided into two parts, namely Dataset 2.1 and Dataset 2.2. In Dataset 2.1, the total number of works is 885, the number of winning works is 240, and the number of works subject to re-evaluation is 28, which is referred to as the small sample dataset. In Dataset 2.2, the total number of works is 9329, the number of winning works is 2977, and the number of works subject to re-evaluation is 342, which is referred to as the large sample dataset. A histogram of the changes in the mean standardized score and range is shown in Figure 4.

(1) Overall Score Variation Analysis of the Two Stages: This study uses the mean of the average standardized scores to describe the overall variation in scores. The mean is the most important statistical feature of the overall score situation, and it is highly sensitive to the trend of change, thus providing a good representation of the overall score variation. By analyzing the small sample dataset and the large sample dataset, we find that their overall variation trends are the same. In the first evaluation stage, the distribution range of the average standardized scores is relatively large, with more than 50% of the works scoring between 40 and 60, showing characteristics of a normal distribution. After the second evaluation stage, the scores are more concentrated in the middle range, and no works have an average standardized score below 30, indicating an overall improvement in the quality of the scores and the level of the works. Additionally, based on the large sample dataset, the overall distribution of scores in the second stage is closer to a standard normal distribution, with a noticeable reduction in the range of the normal distribution compared to the first evaluation stage. The second-stage scores better reflect the true level of the works.
(2) Overall Range Variation Analysis of the Two Stages: In the first stage, more than 80% of the works have a range within 20, with the range showing a tendency to concentrate in the 0–20 range, while it is scattered in the area greater than 20. This is because the score fluctuations were initially processed and standardized during the calculation of the standardized scores. After the second stage, works with a range greater than 20 were completely eliminated (after re-evaluation and adjustment of the range), and the range concentrated in the 0–20 area. However, the range is not completely eliminated, as even during expert re-evaluations, experts’ recognition of different works is not entirely consistent, providing a more precise characterization of the works’ levels.

#### 3.4.2. Specific Implementation of the Range-Based Programmable Model

When establishing the range model, two factors are considered: First, the range model should be able to classify the works, meaning that it can identify works with large ranges based on the scores given by the experts in the first stage and categorize those works with large ranges. Second, the range model should be able to process works programmatically, meaning that for the classified works with large ranges, the model should be able to adjust the scores of these works based on a consistent adjustment principle, resulting in the adjusted scores for the works.

The overall idea of establishing the range model is to train a neural network model using Dataset 2.2. The relationship between the standardized scores in the second stage and the re-evaluated scores is used to determine the relationship between the anomalous expert’s standardized scores, the average standardized scores, and the re-evaluated scores. This relationship is then applied to the processing of anomalous expert scores in the first stage. Specifically, this relationship is applied to Dataset 2.1 for verification.

Specific Implementation Steps of the Range-Based Programmable Model are as follows:

**Step 1: Data Import**. The datasets 2.1 and 2.2 from the problem are imported for further use.
**Step 2: Mean Score Calculation**. The average value $\bar{x_{i}}$ of the standardized scores from the five experts in the first stage of Dataset 2.2 is calculated.
**Step 3: Initial Ranking**. The ranking of the works is determined based on the calculated mean $\bar{x_{i}}$.
**Step 4: Tertile Classification**. Works are classified into three segments: high, medium, and low scores based on their rankings. According to the proportions of works in Datasets 2.1 and 2.2, the top 15% of works are classified as high, the works between 15% and 65% are classified as medium, and the works from 65% to 100% are classified as low. The medium segment refers to the works that are neither high nor low.
**Step 5: Outlier Identification within Middle Segment**. Within the medium segment, a range threshold $\bar{\bar{R}}$ is set. If the range $R_{i}$ of a work *i* exceeds this threshold $\bar{\bar{R}}$, the work is classified as a large range work and extracted. In this study, we choose $\bar{\bar{R}}=20$.
**Step 6: Reassessment Outcome Classification**. Works that undergo re-evaluation in Dataset 2.2 are divided into two categories: those whose re-evaluated scores increase and those whose re-evaluated scores decrease.
**Step 7: BP Neural Network Training**. For each of the two categories, a BP neural network is trained. The independent variables are the standardized scores and the mean of the standardized scores of the experts who need score adjustments, while the dependent variable is the re-evaluated score.

In order to investigate the non-linear relationship between expert standardized scores and re-evaluated scores, an adjustment rule based on standardized scores is provided for the re-evaluated scores. The BP neural network is applied to analyze this relationship for the works that are neither in the high nor low score segments. The data is imported into the MATLAB Neural Network Toolbox, with 70% of the samples used for training, 15% for validation, and 15% for testing. The number of hidden neurons is set to 10, and the training algorithm is the Quantized Conjugate Gradient method.

**Figure 5:**
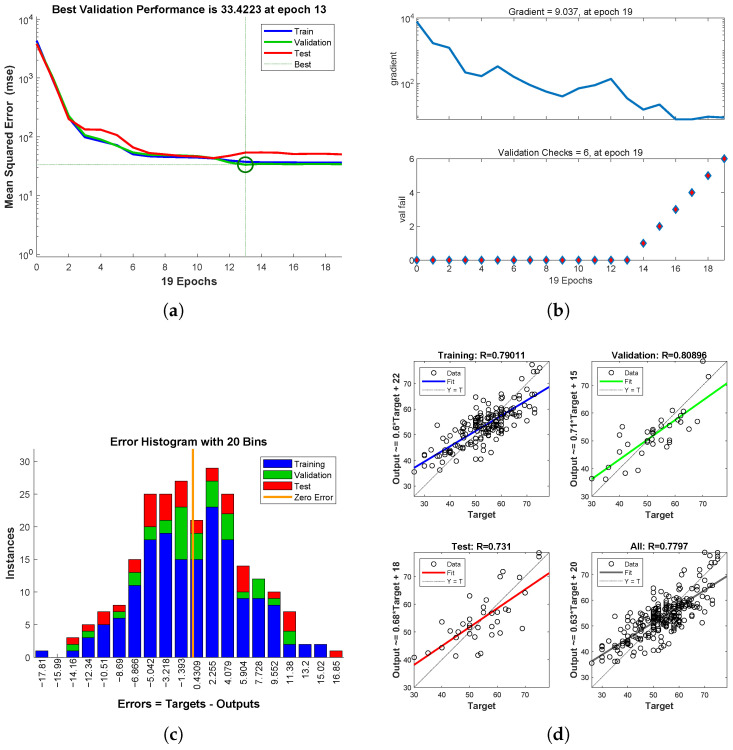
Neural Network Model for Decreased Re-evaluated Scores. (**a**) Mean Squared Error (MSE) Iteration Plot. (**b**) Iteration Count Gradient Change Plot. (**c**) Histogram of Prediction Output and Target Output Errors. (**d**) Fitting Regression.

As shown in Figure 5, for the samples with decreased re-evaluated scores, the training set is trained for 19 iterations. After each training, the parameters of the neural network are adjusted, and the Mean Squared Error (MSE) gradually decreases to a stable value. As shown in Figure 5a, training stops after the MSE of the validation dataset increases consecutively for 13 iterations, with the best model corresponding to the minimum MSE. Figure 5b shows that after 19 training iterations, the MSE stabilizes after the 13th iteration. Figure 5c represents the number of samples in the training set, test set, and validation set that fall within different error values. Figure 5d reflects the regression of the fitted values to the true values, where a higher goodness of-fit indicates better fitting performance.

**Figure 6:**
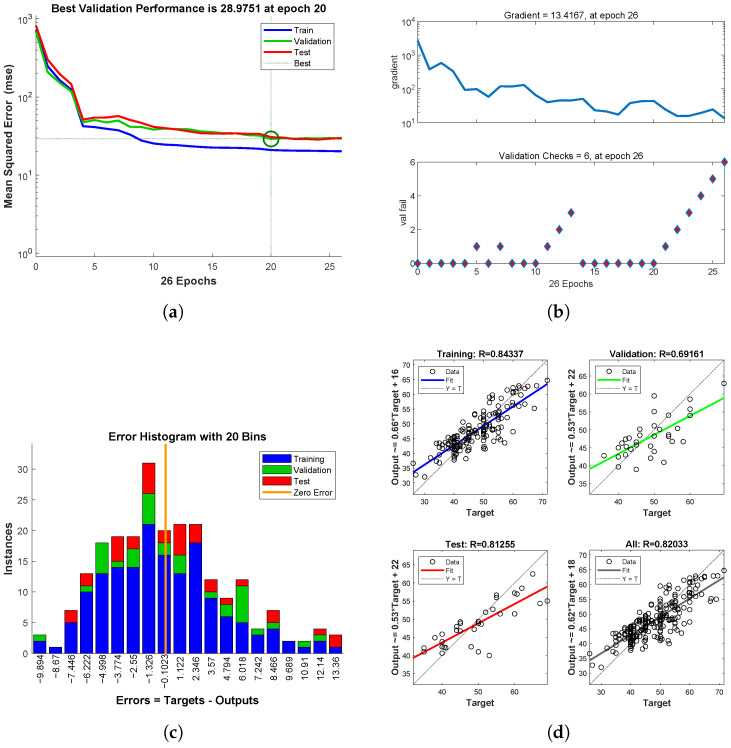
Neural Network Model for Increased Re-evaluated Scores. (**a**) Mean Squared Error (MSE) Iteration Plot. (**b**) Iteration Count Gradient Change Plot. (**c**) Histogram of Prediction Output and Target Output Errors. (**d**) Fitting Regression.

As shown in Figure 6, for the samples with increased re-evaluated scores, the training set is trained for 26 iterations. After each training, the parameters of the neural network are adjusted, and the Mean Squared Error (MSE) gradually decreases to a stable value. As shown in Figure 6a, training stops after the MSE of the validation dataset increases consecutively for 20 iterations, with the best model corresponding to the minimum MSE. Figure 6b shows that after 26 training iterations, the MSE stabilizes after the 20th iteration. Figure 6c represents the number of samples in the training set, test set, and validation set that fall within different error values. Figure 6d reflects the regression of the fitted values to the true values, where a higher goodness of fit indicates better fitting performance.

After the model training is completed, the code for the neural network function is saved, and the works in the up-adjusted and down-adjusted categories are processed and ranked accordingly.

**Step 8: Deviation-Based Classification.** The middle-ranking works extracted from the simulation data 2.1 are divided into two categories: up-adjusted and down-adjusted works. The classification principle is as follows: calculate the absolute difference between the standardized score of the five experts in the first stage and the mean standardized score $\bar{x_{i}}$, and identify the expert corresponding to the maximum absolute difference. If the experts’ score is higher than the mean, the work is classified as down-adjusted; if the experts’ score is lower than the mean, the work is classified as up-adjusted.
  (20) $$l=\max\{x_{i}-\bar{x_{i}}\}$$
**Step 9: Score Adjustment.** For the down-adjusted works, the neural network fitting model for decreased re-evaluated scores is used to adjust the abnormal expert’s standardized scores, resulting in the adjusted re-evaluated scores for the down-adjusted works. For the up-adjusted works, the neural network fitting model for increased re-evaluated scores is used to adjust the abnormal expert’s standardized scores, resulting in the adjusted re-evaluated scores for the up-adjusted works. The results are shown in Table 5 and Table 6.
**Step 10: Final Ranking and Selection.** The up-adjusted and down-adjusted works are re-ranked based on the adjusted re-evaluated scores, and a proportion is determined to select the works that can proceed to the second stage.

The range-based programmable model effectively refined the evaluation process, particularly by handling outliers and inconsistencies in expert evaluations. The neural network’s ability to adjust scores based on re-evaluated results improved the overall reliability and fairness of the evaluation process. This method can implement a programmatic first-stage review process and handle works with large score discrepancies, thereby selecting more innovative works to proceed to the second stage.

### 3.5. Ablation Study and Comparative Experiments

To evaluate the contribution of each component in our proposed framework, we conduct an ablation study with the following model variants:

- **GA + Traditional Z-score**: A baseline model using only the genetic algorithm-based assignment scheme and traditional Z-score for score normalization.
- **GA + Z-score Pro**: Incorporates the improved Z-score Pro model while omitting the BP neural network.
- **GA + Z-score Pro + BP (Full model)**: The complete framework integrating genetic assignment, Z-score Pro adjustment, and the BP-based range-programmable model.

Table 7 presents the performance of each variant on Dataset 2.2.

These results indicate that each model component contributes to performance improvements. Specifically, replacing the traditional Z-score with Z-score Pro yields significant gains in reducing both disorder and divergence. Incorporating the BP-based range programmable model further enhances consistency and alignment with the final reference rankings.

## 4. Conclusions

Currently, the review methods for large-scale innovation competitions have attracted widespread attention. The integration of statistical methods and artificial intelligence technologies has provided valuable insights for scientific reviews. By incorporating entropy-based measures into expert evaluation modeling, the proposed framework not only detects outliers more effectively but also enhances fairness and consistency in final decision-making. In order to select more innovative works in large-scale innovation competitions and conduct the review process scientifically, this study proposes a review scheme based on the Z-score Pro calculation model and BP neural network model. The proposed method can be widely applied to the evaluation processes of large-scale competitions, such as mathematical modeling contests and innovation design competitions. These competitions typically not only demand high-quality work but also place significant emphasis on innovation, which aligns well with the design principles of our methodological framework.

This method provides a reference for the review of such competitions, but there are many factors influencing the actual review process. The review results are affected by various factors, including but not limited to expert preferences, the experts’ familiarity with the works, and the number of review stages. The interaction of these factors requires a more comprehensive model. Future work will involve the development of a more adaptable scheme. Therefore, we propose several perspectives for future research:

(1) Expert Factors Study: The review process in the real world is multi-stage and complex. In the early stages of group decision-making, experts’ opinions often conflict or show significant disagreement. At this point, interaction between experts is required to reach a consensus. Additionally, experts have different preferences and varying degrees of familiarity with works in different fields. Future research may include studies on the group decision-making process and consensus-building among experts, considering expert preferences and familiarity in the review process.
(2) Application of Artificial Intelligence Methods: Currently, artificial intelligence technologies are developing rapidly. For large-scale competition reviews, the number of works is large in the early stages, requiring significant time and cost for expert reviews. Moreover, different experts have different review criteria. Future research may explore the application of more advanced artificial intelligence methods in the early stages of the review process to propose a more scientific and programmatic review scheme.
(3) Despite the promising results, it is important to acknowledge the limitations of the current dataset. The experimental data, while representative within its scope, may not fully capture the diversity of real-world evaluation contexts. Variations in institutional practices, expert behavior, and contextual dependencies could affect the generalizability of the proposed framework. Further validation on broader datasets is, therefore, necessary to confirm the robustness and scalability of the model across different application scenarios.

## Figures and Tables

**Table 1:** Correlation between Evaluation Metrics and Ranking Deviation (Kendall’s Tau Distance).

Metric	Spearman’s ρ	*p*-Value
Overlap Degree	−0.76	<0.01
Disorder Degree	0.82	<0.01
Divergence Degree	0.69	<0.05
Award Change Degree	0.58	<0.05

**Table 2:** Expert Reviewers Assigned to Selected Works.

Work ID	Expert Reviewer ID				
Work 1	68	115	28	17	8
Work 2	107	14	94	55	48
Work 3	115	1	116	113	26
Work 4	44	72	71	116	97
Work 5	35	24	111	78	54
…					
Work 2996	29	60	44	84	32
Work 2997	117	58	7	34	5
Work 2998	71	86	13	89	84
Work 2999	42	50	91	70	3
Work 3000	47	98	69	113	43

**Table 3:** The rankings of the 27 first-prize works calculated using the Modified Z-score calculation model and the Z-score Pro model.

Original Rank	Modified Z-Score Rank	Z-Score Pro Rank	Original Rank	Modified Z-Score Rank	Z-Score Pro Rank
**1**	1	1	**15**	44	15
**2**	7	5	**16**	54	34
**3**	157	180	**17**	78	66
**4**	29	10	**18**	19	23
**5**	30	26	**19**	180	140
**6**	197	135	**20**	106	300
**7**	65	21	**21**	102	123
**8**	77	55	**22**	42	4
**9**	60	78	**23**	359	291
**10**	35	31	**24**	144	166
**11**	125	80	**25**	22	29
**12**	51	41	**26**	87	63
**13**	2	8	**27**	182	150
**14**	305	106			

**Table 4:** Three Metrics Calculated Using Two Methods for 27 First-Place Works.

Method	Overlap	Disorder	Divergence
**Modified Z-score**	1	2210	119.85
**Z-score Pro**	2	1849	106.80

**Table 5:** Adjusted Re-evaluation Scores for the Downward-Tier Works.

Original Ranking	Expert	Original Score	Re-Evaluation Score
**40**	P493	66.88	54.69768143
**109**	P117	78.11	57.17969894
**199**	P260	70.71	53.99263382
**221**	P253	71.84	56.96060562
**478**	P183	65.39	49.39115143
**451**	P280	69.46	49.82651138
**357**	P125	66.83	51.74188995
**448**	P530	66.01	49.95048141
**323**	P260	67.87	52.43124008
**341**	P253	68.29	52.03367996
**527**	P394	61.99	48.38467789
**491**	P125	61.84	49.18770599
**432**	P125	61.84	50.33391953
**343**	P262	65.12	52.04156876
**548**	P322	60.35	48.03154755
**468**	P372	63.08	49.71671677
**289**	P117	69.24	52.96650314
**283**	P255	66.87	53.07575607
**266**	P434	69.14	53.30323792
**248**	P301	74.28	53.74472809
**260**	P440	73.09	53.38253021

**Table 6:** Adjusted Re-evaluation Scores for the Upward-Tier Works.

Original Ranking	Expert	Original Score	Re-Evaluation Score
**32**	P378	43.96	48.14201355
**50**	P308	40.86	46.07950974
**85**	P117	43.44	47.13598251
**155**	P117	43.44	47.93474579
**196**	P370	40.63	46.29893494
**235**	P370	44.49	47.53723526
**375**	P117	41.02	44.14457321
**403**	P407	29.61	39.84391403
**465**	P117	38.6	42.22123337
**458**	P260	38.04	41.978508
**537**	P260	35.2	39.84025574
**504**	P037	37.54	41.27183151
**392**	P345	36.89	41.97652435
**526**	P260	35.91	40.27737045
**305**	P117	43.44	45.99944687
**307**	P434	42.56	45.55607224
**250**	P154	41.63	45.56154633
**269**	P460	39.57	44.28785324
**273**	P625	41.06	45.00710678

**Table 7:** Performance Metrics for Different Model Variants.

Model Variant	Disorder Degree ↓	Divergence ↓	Overlap Degree ↑
GA + Traditional Z-score	2311.5	127.60	0
GA + Z-score Pro	1849.2	106.80	2
GA + Z-score Pro + BP (Full)	**1730.4**	**98.45**	**4**

## Data Availability

The data presented in this study are available on https://github.com/yuyu-2019/data (accessed on 13 May 2025).
